# Tomato flu in children in India: What evidence do we have so far?

**DOI:** 10.1002/puh2.35

**Published:** 2022-11-14

**Authors:** Parimala Mohanty, Prakasini Satapathy, Vanessa Jaëlle Dor, Diana Androga, Aroop Mohanty, Bijaya Kumar Padhi, Ranjit Sah

**Affiliations:** ^1^ Department of Community Medicine Institute of Medical Sciences & SUM Hospital Siksha ‘O’ Anusandhan Deemed to be University Bhubaneswar India; ^2^ Department of Virology Postgraduate Institute of Medical Education and Research Chandigarh India; ^3^ Harvard Medical School Boston USA; ^4^ London School of Hygiene and Tropical Medicine London UK; ^5^ All India Institute of Medical Sciences Gorakhpur India; ^6^ Department of Community Medicine and School of Public Health Postgraduate Institute of Medical Education and Research Chandigarh India; ^7^ Institute of Medicine Tribhuvan University Teaching Hospital Kathmandu Nepal

**Keywords:** India, outbreak, tropical infection, viral exanthem

Dear Editor,

A rare, highly contagious viral infection known as “tomato flu” is spreading among children in India [1, 2]. After the first patient was diagnosed in May 2022 in the southern state of Kerala, tomato flu has been reported in over 100 young children by August in Kerala, Tamil Nadu, Odisha, and Haryana [3]. The emergence of increasing cases in the initial phases has raised concerns regarding the rapidity of its spread. According to experts, young children are most vulnerable to this illness because their immune systems are underdeveloped, making them more prone to becoming infected, but it does not easily pass on to adults [4].

Another possible factor contributing to the increase in infection among young children is the reopening of schools following their closure during the COVID‐19 pandemic. It is seen that children in the age group of 1–9 years are mostly affected by the outbreak. Though children as old as 9 years have been affected, it is reported that the virus is mostly seen in children under‐5 years. In the United Kingdom, the first cases described were that of a 5 year boy and his infant sister (13 months). They had rashes on their hands and legs 1 week after returning from a family vacation in Kerala, India. They denied interacting with sick children, despite having played with another child who had recently recovered from “tomato flu” like symptoms [3, 4].

Instances similar to tomato fever were previously recorded in 2007 in Kerala, when many people were affected in Varzur, Mudakayam, and Kanirapally in Kottayam and Pathinamtita districts, where chikungunya was also reported at the same time. However, the increasing cases of tomato flu have now become a concern as it is found to affect more children under five than usual [1]. Tomato flu infection is believed to be a variant of the viral hand‐foot‐and‐mouth disease (HFMD), a common condition primarily affecting children aged 1–5 years [2]. According to the Centers for Disease Control and Prevention, HFMD is a highly contagious disease caused by the coxsackievirus (CA16) and spreads quickly through an infected person's nasal and throat secretions, excrements, and fluid from blisters. Fever and flu‐like symptoms, oral sores, and skin rash are common symptoms. The CA16 is one of India's most common enteroviruses and one should not expect this infection to be found limited to a single geographic region [5].

This viral infection is known as tomato flu because the symptoms include little grapelike blisters that can grow as large as a tomato and are red like a tomato. It has nothing to do with tomatoes or the use of tomatoes. The primary symptoms in children include fever, sores in the mouth, a skin rash, fatigue, vomiting, nausea, dehydration, body aches, intense joint pain, and diarrhea. Traditionally, tomato flu rashes were limited to the mouth (tongue, gums, and inside of the cheek), palms, and soles. However, clinicians are now reporting rashes on the buttocks and peeling of the nails. The tomato flu is a non‐life threatening viral infection with symptoms similar to coronavirus or monkeypox infections [3]. In light of the COVID‐19 pandemic, increased advancements in surveillance and reporting systems may have picked up the occurrence of this infection. However, it is possible that the long‐term effects of COVID‐19 could also have led to an increase in infections in children with a weaker immune system. The other symptoms of the disease include fatigue, nausea, vomiting, dehydration, joint swelling, diarrhea, fever, body aches, and frequent influenza‐like symptoms similar to those seen in dengue or chikungunya. The blisters are also claimed to mimic those observed in people infected with the monkeypox virus. Laboratory testing for tomato flu is performed in children with symptoms to rule out chikungunya, dengue, varicella‐zoster virus, zika virus, and herpes; once these viral diseases are ruled out, tomato virus infection is confirmed [4].

Following the surge of this new virus strain, which generally flared up in nursery schools and daycare centres, India's Ministry of Health and Family Welfare has issued an advisory on the management of the disease. As a precautionary step, the Ministry has directed the states to conduct proper screening and increased surveillance. Those infected with this flu symptoms are advised isolation for 5–7 days from the onset of symptoms to prevent the spread of infection to other children. To prevent flu transmission, utensils, clothing, and other items used by infected people must be sanitized. Children are advised to maintain good personal hygiene, avoid thumb or finger sucking, not hug or come in contact with other children who have fevers or rashes, and use a handkerchief if they have a runny nose. Most importantly, if one detects any of the symptoms mentioned above, they should get medical attention. Tomato flu is a self‐limiting infection and can be managed with symptomatic treatment. It is recommended that testing be done if an outbreak occurs in that particular geographic area.

The possibility of an entirely new virus as “tomato flu” has been refuted by recent studies [6, 7]. However, the peculiar presentation of clinical phenotypes could demonstrate the role of mutation [2]. The available scientific information is inconclusive in nature. Thus genotyping of clinical cases should be prioritized. Since no specific drug exists to treat the disease, health‐promoting activities should be implemented to contain the virus. Special attention should be warranted to vulnerable communities such as people living in regions where dengue, chikungunya, and malaria are endemic.

## AUTHOR CONTRIBUTIONS

Parimala Mohanty: Conceptualization, Data curation, Writing – original draft. Prakasini Satapathy: Conceptualization, Methodology, Writing – review & editing. Diana Androga: Writing – review & editing. Bijaya Kumar Padhi: Conceptualization, Supervision, Writing – review & editing. Ranjit Sah: Writing – review & editing.
